# Conversion of a sequential inductively coupled plasma emission spectrometer into a multichannel simultaneous system using a photodiode array detector

**DOI:** 10.1155/S146392469800008X

**Published:** 1998

**Authors:** Maria Fernanda Pimentel, Mário César Ugulino Araújo, Benício de Barros Neto, Célio Pasquini

**Affiliations:** 1 Fundação Instituto Tecnológico do Estado de Pernambuco ITEP Av. Prof. Luís Freire, 700 Cidade Universitária Recife PE 50740-540 Brazil; 2 Departamento de Química CCEN Universidade Federal da Paraíba João Pessoa PB Brazil; 3 Departamento de Química Fundamantal CCEN Universidade Federal de Pernambuco Recife PE 50739-907 Brazil; 4 Instituto de Química Universidade Estadual de Campinas UNICAMP Campinas SP Brazil

## Abstract

A monochannel plasma emission spectrometer was converted to a multichannel instrument by the introduction of a detection system based on an array of 1024 photodiodes and a low-resolution dispersion device. The new, relatively inexpensive equipment, features both the high speed typical of simultaneous instruments and the versatility of scanning systems. This paper reports on an evaluation of the modified equipment for quantitative analysis with the simultaneous determination of Al, Mn, Mg, Ca, Fe and Cu in a natural water matrix. An average relative prediction error of 2.4% was found which is the same as the error obtained with the conventional analytical method. Data acquisition with the modified instrument is up to 40 times faster.


Journal of Automatic Chemistry, Vol. 20, No. 3 (May-June 1998) pp. 69-75

### Conversion of a sequential inductively coupled plasma emission spectrometer into a multichannel simultaneous system using a photodiode array detector

**Maria Fernanda Pimentel***

*Fundação Instituto Tecnológico do Estado de Pernambuco, ITEP, Av. Prof. Luís Freire, 700, Cidade Universitária, 50740-540, Recife, PE, Brazil*

**Mário César Ugulino de Araújo**

*Departamento de Química, CCEN, Universidade Federal da Paraíba, João Pessoa, PB, Brazil*

**Benício de Barros Neto**

*Departamento de Química Fundamental, CCEN, Universidade Federal de Pernambuco, 50739-907, Recife, PE, Brazil*

**and Célio Pasquini**

*Instituto de Química, Universidade Estadual de Campinas, UNICAMP, Campinas, SP, Brazil*

*A monochannel plasma emission spectrometer was converted to a multichannel instrument by the introduction of a detection system based on an array of 1024 photodiodes and a low-resolution dispersion device. The new, relatively inexpensive equipment, features both the high speed typical of simultaneous instruments and the versatility of scanning systems. This paper reports on an evaluation of the modified equipment for quantitative analysis with the simultaneous determination of Al, Mn, Mg, Ca, Fe and Cu in a natural water matrix. An average relative prediction error of 2.4% was found which is the same as the error obtained with the conventional analytical method. Data acquisition with the modified instrument is up to 40 times faster.*

### Introduction

Inductively coupled plasma atomic emission spectrometry (ICP-AES) has become a very popular technique for elemental analysis in environmental studies, petrochemistry, metallurgy and several other applications, with annual sales figures for ICP spectrometers estimated at 1000 instruments internationally [1]. The reasons for such success may be ascribed to the low detection limits, relatively small inter-element matrix effects, wide linear dynamic ranges, high precisions (0.5–5%) and the ability to perform simultaneous multicomponent analyses that are characteristic of ICP spectrometry [2].

Although the amount of information that can be generated simultaneously by an ICP source is enormous, many of the commercially available instruments do not take advantage of it, because they use detection systems based on photomultiplier tubes (PMT). The most common configurations correspond either to sequential or direct reading instruments. In sequential systems, light dispersion is usually done by either a holographic grating monochromator or a conventional ruled grating. Detection is performed with one or two photomultiplier tubes that can acquire information only in a narrow bandwidth at a time, therefore requiring relatively long periods of time for data recording over several wavelengths. Because of their excellent flexibility, these instruments are more useful for research applications. Direct-reading spectrometers, on the other hand, usually contain a single polychromator with several fixed exit slits and photomultipliers placed on purpose to register the spectral lines of interest. Although capable of monitoring wide spectral ranges, and thus allowing simultaneous multicomponent analysis, they suffer from geometrical and mechanical constraints imposed by the dimensions of a PMT that limit the minimum distance between two analytical lines monitored in the same polychromator. Furthermore, there is no flexibility in line selection; the instrument is not easily reprogrammed to record a new set of analytical lines, and costs increase steeply with the number of lines monitored. Such instruments are best suited to high-volume runs under routine protocols, for samples of known approximate composition.

Ideal detectors for atomic emission spectroscopy would combine the PMT advantage of direct light conversion into an analogue response with the ability to simultaneously detect the entire spectrum that is exhibited by the old photographic plates [3]. To find a multidetection system that meets these requirements many different sensors have been investigated, such as vidicon [4], photodiode arrays (PDA) [5–14] and charge transfer devices (CTD) [15–19], which include charge injection devices (CID) and charge-coupled devices (CCD).

CTD detectors, normally found in bidimensional shapes, are adequate for coupling with dispersion systems using echelle gratings [15, 16]. The high cost of these sensors has limited their use, but they could play an important role in the future—some companies have begun to sell plasma emission spectrometers based on these devices [1, 18, 19]. Photodiode arrays, on the other hand, have become very attractive as multichannel detection devices for atomic spectrometry instruments, because of their low cost and ease of operation [6, 7].

The applicability of unidimensional image sensors to simultaneous multichannel spectroscopy hinges on the compromise, imposed by the finite width of these detectors, between spectral resolution and the extent of the spectral window that can be monitored simultaneously. The need for high spectral resolution is due mainly to the choice of traditional univariate methods for data proces-

* Author to whom correspondence should be addressed.

0142-0453/98 $12·00 © 1998 Taylor & Francis Ltd sing. Now that microcomputers are available in laboratories, multivariate methods are becoming increasingly popular in ICP-AES, allowing all the available spectral information to be processed at the same time and minimizing overlapping problems [20–26]. With multivariate analysis, low-resolution dispersion systems can be used even for complex matrices where interference-free spectral lines cannot be found.

This paper describes the conversion of a monochannel plasma emission spectrometer into a multichannel instrument. A detection system based on an array of 1024 photodiodes and a low-resolution dispersion device was added. The new, relatively inexpensive equipment, features both the high speed typical of simultaneous instruments and the versatility of scanning systems.

### Experimental

### *System description*

The scheme for converting the commercial monchannel spectrometer (a Spectroflame model from Spectro Co.) into a multichannel instrument is shown in figure 1. Of the original Spectroflame equipment, the final configuration retains only the light source and the sample introduction device.

Radiation from the plasma source is led through an optical fibre (Spectro, quartz) to the entrance slit of the dispersion system, which is 120 μm wide. Since the original equipment already had a support for optical fibres close to the torch, no internal modifications were needed.

The dispersion system, manufactured by Oriel (model 77200), has an asymmetrical Czerny-Turner configuration with a 0.25-m focal distance that allows easy exchange of diffraction gratings. The multidetection system is placed in its output focal plane and mounted inside an aluminium box carefully insulated against external light. The photodiode array is an EG&G Reticon RL1024SAQ model included in a 22-pin chip, with a working area 25.4 mm wide and 2.5 mm high. This device, containing 1024 photodiodes, is sealed with a quartz window and exhibits a spectral response in the range 200–1000 nm.

The video signals generated by the PDA are analogically processed by two printed circuit boards, also manufactured by Reticon. The motherboard, RC1000, measures 20 cm × 10 cm. The sensor is mounted on the satellite board RC1001, whose dimensions are 10 cm × 5 cm. A hole in the board underneath the PDA allows connection to the cooling system described below.

The motherboard has a complete internal timing system that allows operation of the PDA without external interface. The boards can also be made to accept start and clock external signals, if the RC1000 jumpers are changed to prevent access of the internal oscillators and integration counters. In the present work, the start and clock signals were generated by software that makes the synchonization procedure and the selection of integration times easier to carry out. The PDA internal timing system was employed only in the video signal synchronizing procedure recommended by the manufacturer [27].

In addition to the photodiode array and its control boards RC1000 and RC1001, the aluminium box is equipped with an XYZ displacement support and the PDA cooling system.

To perform acquisition of the signals generated by the photodiode array and transfer them to a microcomputer, as well as sending to the PDA the control signals originated by the control software, a parallel interface was developed.

The system for simultaneous multidetection presented in this paper is based on the system reported by Belatto *et al.*, who used it with a plane grating spectrograph [14]. The details of its construction are given in the following sections.

### *The PDA cooling system*

The PDA dark current is temperature dependent; a 10° temperature reduction decreases the dark current by approximately 50% [28]. To minimize the dark current and so allow an integration time of a few seconds to be employed, a cooling system based on four Peltier elements (RS Components, RS618-718) was coupled to the photodiode array [29].

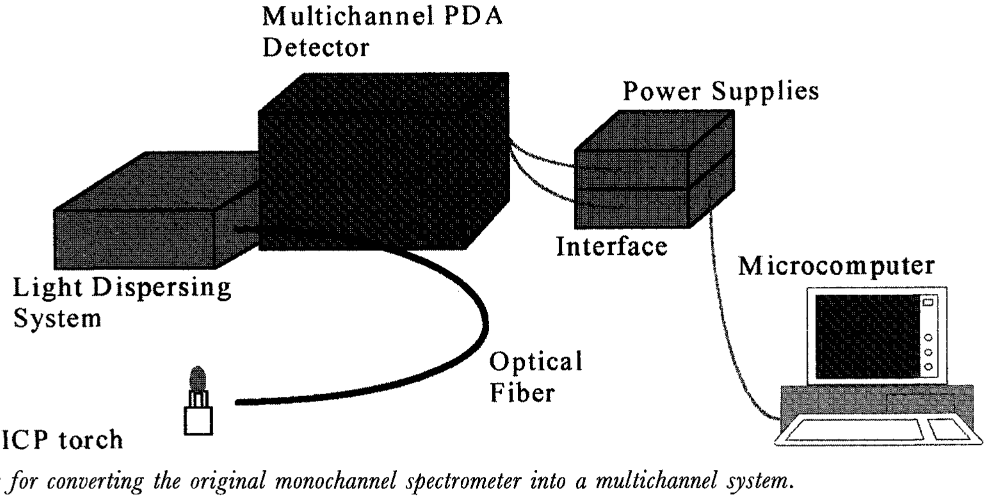

*Figure 1. Scheme for converting the original monochannel spectrometer into a multichannel system.*

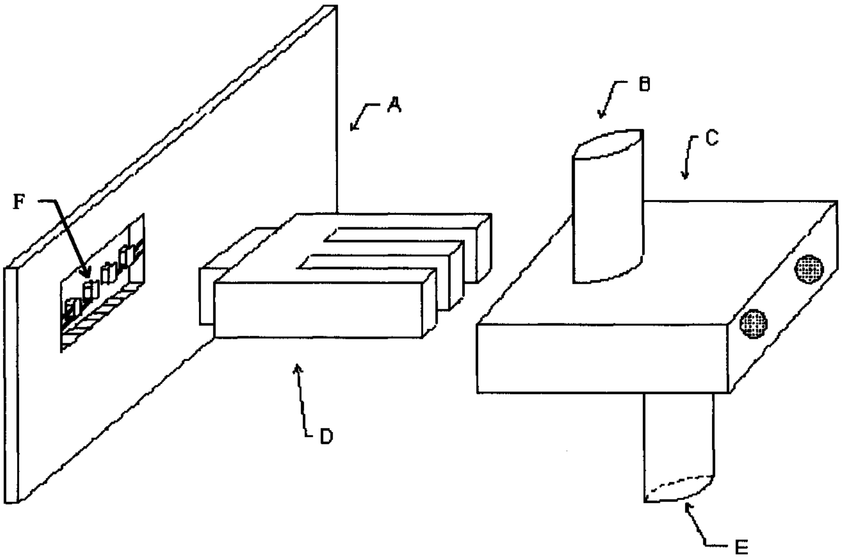

*Figure 2. Blown-up view of the heat exchanger. A = satellite board; B = air in; C = acrylic box; D = copper block; E = air out; F = Peltier elements.*

A Peltier element functions as a heat pump, transferring heat from one of its faces to the opposite one, where the heat is dispersed through contact with a copper block acting as a heat exchanger. The block is mounted inside an acrylic box with forced air circulation, as shown in figure 2.

The Peltier elements are pressed against the lower part of the PDA by the heat exchanger. A thermal paste was smeared onto the interfaces to help fasten the Peltier elements in position and to secure efficient thermal contact. For an integration time of 1 s this arrangement allowed an average reduction of approximately 35% in the background signal intensity.

### *Setting up the multidetection system*

The satellite board containing the PDA and the cooling system are mounted on an XYZ translating support that allows the entire set to be moved in micrometric displacements. This motion is needed to adjust the detector position relative to the monochromator focus. A protective plate is placed in front of the PDA, to prevent damage during focus adjustment. Figure 3 shows a side view of the whole PDA-cooling system-XYZ translator apparatus.

The aluminium box where the multidetection system is mounted is 21 cm wide, 40 cm high and 30 cm deep. To reduce noise levels, the control boards RC1000 and RC1001 are placed so as to minimize the length of the signal transmission cables between them (particularly the analogue signal transmission cable). Flexible plastic tubes are used to carry air from the pump to the aluminium box, where it circulates inside latex tubing.

### *Interface*

A parallel interface is used to acquire the data and transfer them to the microcomputer and also to send the software-generated control signals to the PDA. This interface has a 12-bit analogue-to-digital converter (AD 7672KN-3) fed by a very precise reference power source (AD-584) [see 14, 30, 31].

### *Software*

The software Plasma, written in QuickBasic 4.5, generates the start and clock signals, performs data acquisition and handling and saves and displays the recorded spectra. The flowchart on which the software is based is shown in figure 4. Its main subroutines can be found in Belatto *et al.*[14].

### *Focus adjustment*

A helium–neon laser (632.8 nm) was employed for the initial adjustment of the PDA position with respect to the dispersion system focus. The diffraction grating (1200 grooves/mm) was displaced until the 632.8 nm line fell in the centre of the output focal plane. This was done by turning a small handle located on the side of the dispersion system until the selected wavelength was shown in a dial. Turning the micrometric screws of the XYZ displacement system, the satellite board containg the PDA was moved little by little, and after each small displacement the radiation emitted by the laser was observed. The adjusting phase was finished after a peak of satisfactory width and maximum intensity was being read by photodiode number 512, located in the centre of the array.

To verify the adjustment in another spectral range and also to perform a finer focus adjustment, the spectrum of a standard 2 mg/l manganese solution was employed. The diffraction grating was placed so that the dial of the dispersion system read 257.61 nm, indicating that this particular wavelength would be monitored by diode number 512. The detector was then displaced by small rotations of the screws, with the goal of obtaining the best possible separation between the manganese emission lines at 257.61 nm, 259.373 nm and 260.569 nm, but taking

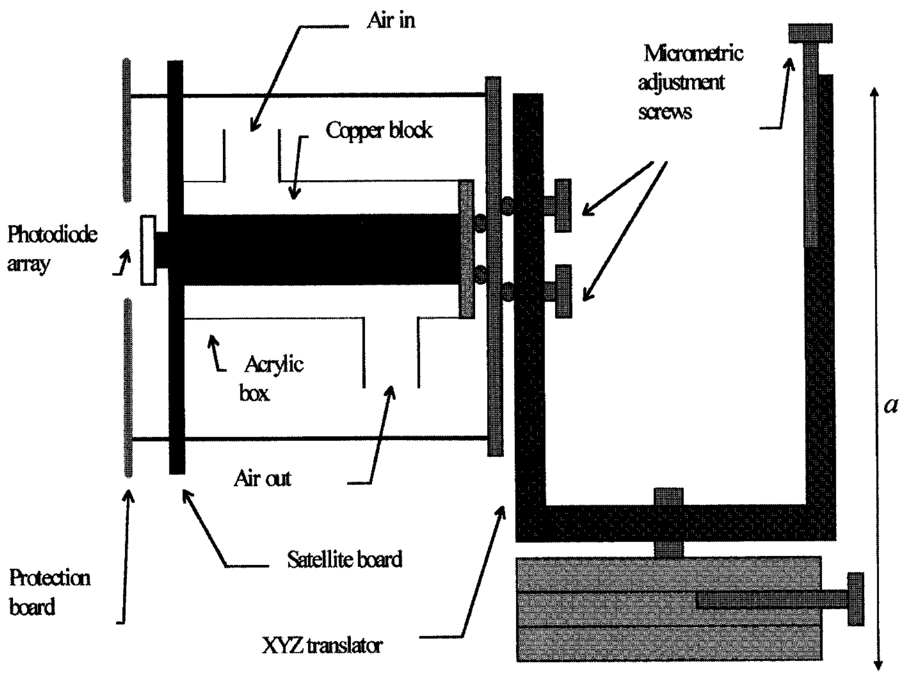

*Figure 3. Side view of the XYZ translation system and of the heat exchange set;* a = *13 cm.*

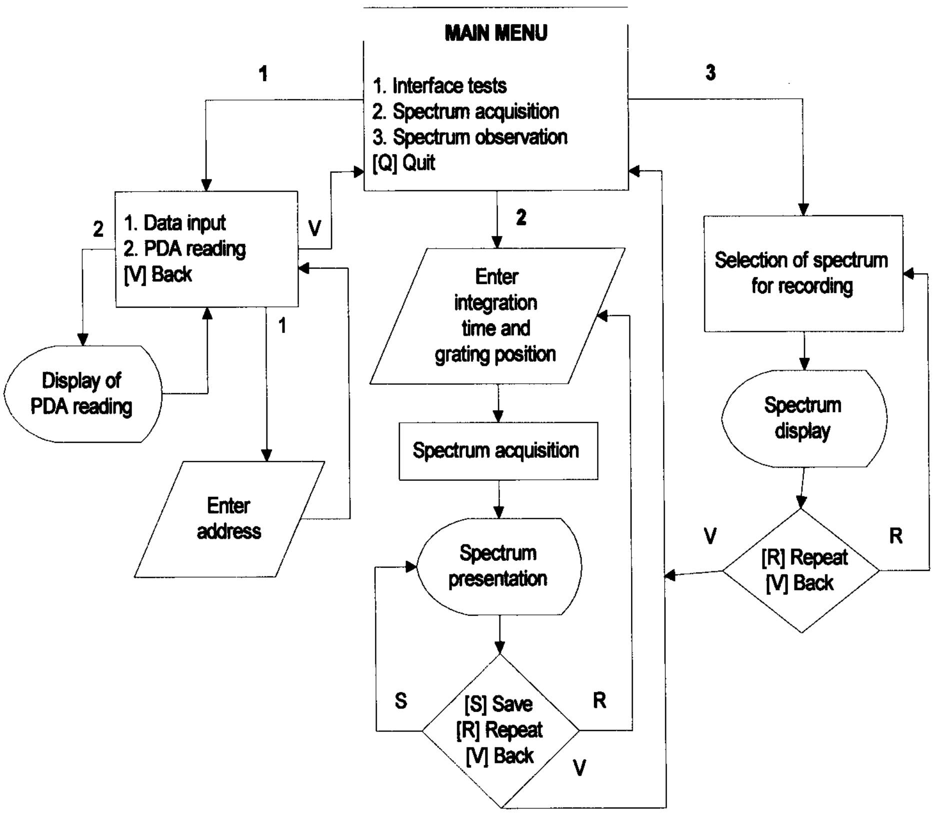

*Figure 4. Flowchart for the software Plasma, the control signal generation and data acquisition program.*

care to keep the 257.61 nm line at the centre of the photodiode array.

*Optical calibration*

To find the ratio nm/diode an optical calibration was performed with spectra of standard solutions of manganese, cadmium, nickel and chromium recorded in several windows with the 1200 grooves/mm grating.

To perform the calibration, the most intense peaks are identified in each spectrum and the wavelength corresponding to the maximum of each peak is related to the respective photodiode. Least-squares fitting of a straight line to the wavelength versus photodiode data then yields the ratio 0.079 nm/diode. Since there are 1024 diodes in the array, the wavelength range covered simultaneously is 0.0791 nm/diode × 1024 diodes = 81 nm.

Although only the 1200 grooves/mm diffraction grating was used, exchange of gratings is very simple, allowing easy modification of the dispersion and consequently of the wavelength range covered simultaneously.

### Results and discussion

The capability of the modified equipment for quantitative analysis was evaluated with simultaneous determinations of aluminium, manganese, magnesium, calcium, iron and copper in matrices of natural waters. For the simultaneous determination of these six elements a spectral window covering the 250–331 nm range is the most convenient. The individual spectral lines, chosen on the basis of their intensities and absence of spectral overlapping, are the following: 263.1 nm for iron, 285.2 nm for magnesium, 317.9 nm for calcium, 398.2 nm for aluminium and 324.2 nm for copper.

The experimental design for the calibration step, i.e., the choice of concentration levels and of the number of replicates in each level was done as recommended [32]. Multielemental standard solutions were prepared with four concentration levels, and three authentic replicates were made at both ends of the concentration range, to allow a lack-of-fit F test of the linear models [33]. The final concentrations were the following (in mg/l):

Ca, Al and Fe: 2.0, 2.0, 2.0, 4.0, 8.0, 10.0, 10.0, 10.0.

Mn and Cu: 0.5, 0.5, 0.5, 1.0, 2.0, 3.0, 3.0, 3.0.

Mg: 1.0, 1.0, 1.0, 2.0, 4.0, 5.0, 5.0, 5.0.

*Table 1. Operating conditions of the ICP spectrometer.*

| | |
|---|---|
| Argon flow | 12 l min$^{-1}$(coolant)<br>0.5 l min$^{-1}$ (auxilary)<br>1.0 l min$^{-1}$ (nebulizer) |
| Sample uptake rate | 2.3 l min$^{-1}$ |
| RF power | 1 kW |
| Observation height | 15 mm above the coil |
| Integration time | 1.5 s |

*Table 2. Calibration results for the six elements, with standard errors (in parentheses).* a *and* b *are respectively the linear and angular coefficients in the fitted models.* $V_{expl}/V_{max}$ *is the ratio between the variance explained by the model and the maximum explainable variance.*

| Element | *Parameter values (standard errors)* a | b | $V_{expl}/V_{max}$ |
|---|---|---|---|
| Cu | −27.2 (±9.2) | 619.4 (±4.6) | 99.99 |
| Mn | −3.3 (±13.1) | 1065.7 (±6.4) | 99.99 |
| Fe | 6.2 (±11.3) | 80.3 (±1.6) | 99.92 |
| Mg | 26.0 (±21.6) | 555.2 (±6.2) | 99.99 |
| Ca | −4.1 (±6.4) | 119.5 (±0.9) | 99.99 |
| Al | 6.3 (±12.0) | 117.5 (±1.7) | 99.94 |

Both these solutions and the synthetic test samples were prepared by dilution of Tritimex ampoules (Merck) with water newly purified by the Milli-Q system (Millipore).

The operating conditions of the spectrometer are shown in table 1. Although in the present application a single (1.5 s) integration time was employed, multiple integration times can be used by widening the working dynamic range [34].

Figure 5 present the spectra of two multielemental calibration solutions with concentrations of all elements corresponding to the lower and upper limits of the calibration ranges. The working spectral lines are also indicated.

The calibration curves were determined by least-squares fitting of the model $I = a + bC$ (where $I$ and $C$ stand for an intensity and its corresponding concentration, respectively) to the calibration data of each element. The resulting parameter values are shown in table 2. No lack of fit was detected by the F test for any model, at the 95% significance level. The excellent fit of the linear models was confirmed by the ratio between the explained variance and the maximum explainable variance, which exceeded 99.9% in all cases.

To test model accuracy, three synthetic samples simulating matrices of natural water, prepared in duplicate, were analysed with the modified instrument and the calibration parameters shown in table 2. The nominal concentrations of the three test samples are the following (in mg/l, in the order Mn, Fe,Mg, Al, Ca, Cu):

Sample 1: 1.0 5.0 1.5 9.0 3.0 2.5.

Sample 2: 2.5 3.0 3.0 3.0 5.0 1.0.

Sample 3: 2.0 7.0 4.5 7.0 9.0 1.5.

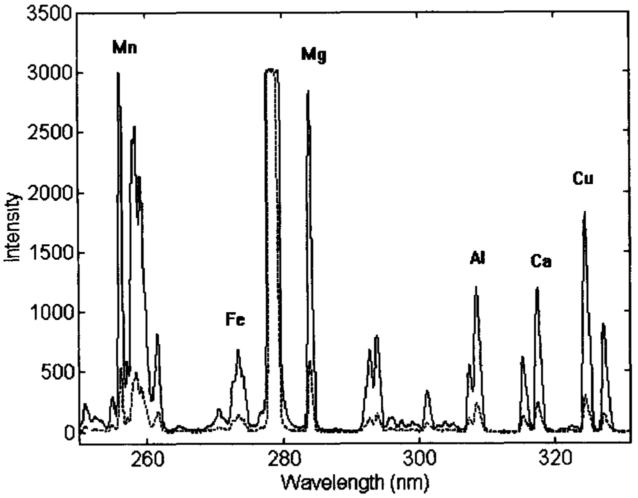

*Figure 5. Spectra of standard calibration solutions with concentrations corresponding to the upper and lower limits of the concentration range (full and dashed lines, respectively). The working spectral line of each element is indicated by the corresponding chemical symbol.*

*Table 3. Comparison of the concentrations predicted by the two methods for each element in the three duplicate synthetic test samples. Std and Mod refer to the standard and to the modified equipments, respectively. Concentration units are mg/l. Values in parentheses are the relative errors given by* $(C_p - C_e)/C_e) \times 100$, *where* $C_p$ *and* $C_e$ *stand for predicted and expected concentrations, respectively. In the two last columns the relative errors for each element are averaged over the samples for both methods.*

| | *Sample 1* | | *Sample 2* | | *Sample 3* | | *Average relative error, %* | |
|---|---|---|---|---|---|---|---|---|
| *Element* | *Std* | *Mod* | *Std* | *Mod* | *Std* | *Mod* | *Std* | *Mod* |
| Mn | 1.05 (+5.0) | 1.04 (+4.0) | 2.40 (−4.0) | 2.59 (+3.6) | 2.00 (−2.0) | 2.06 (+3.0) | 3.7 | 3.5 |
| Fe | 4.80 (−4.0) | 5.04 (+0.8) | 3.13 (+4.3) | 3.05 (+1.7) | 6.90 (−1.4) | 6.99 (−0.1) | 3.2 | 0.9 |
| Mg | 1.54 (+2.7) | 1.56 (+4.0) | 3.06 (+2.0) | 3.10 (+3.3) | 4.54 (+0.9) | 4.57 (+1.5) | 1.9 | 2.9 |
| Al | 8.89 (−1.2) | 8.89 (−1.2) | 3.00 (0.0) | 2.96 (−1.3) | 6.78 (−3.1) | 6.79 (−3.0) | 1.4 | 1.8 |
| Ca | 2.89 (−0.7) | 3.06 (+2.0) | 5.03 (+0.6) | 5.08 (+1.6) | 8.81 (−2.1) | 8.95 (−0.6) | 1.1 | 1.4 |
| Cu | 2.58 (+3.2) | 2.59 (+3.6) | 1.00 (0.0) | 0.98 (−2.0) | 1.41 (−6.0) | 1.41 (−6.0) | 3.1 | 3.9 |
| | | | | | Overall relative error (%) | | 2.4 | 2.4 |

The same samples were also analysed conventionally, to provide a comparison of the method proposed here with the original detection system of the Spectroflame equipment.

The concentration values predicted by the two methods for the six test samples are given in table 3, along with the percent errors relative to the expected values. Although there are differences in the errors from element to element, the average error over all the analyses is by coincidence exactly the same for both methods: 2.4%. In the modified multidetection method, however, the intensity values for all six elements in a sample are acquired in only 1.5 s, while the original PMT scanning system requires 65 s (with off-peak background correction).

### Conclusions

As well as offering the high speed typical of simultaneous multidetection spectrometers, the system proposed in this paper also exhibits the characteristic versatility of scanning systems. In the application reported here a 1200 grooves/mm grating was used, recording simultaneous spectra in an 81-nm window between 250 nm and 331 nm but, for another application, a different window could easily be chosen. Exchange of the diffraction grating also allows easy modification of the width of the spectral range covered by the system. For complex matrices, where lines free from spectral interference cannot be found due to the low resolution of the dispersion system, multivariate calibration techniques like PLS and PCR can be adopted [35].

Another significant feature of the PDA system is its comparatively low cost. It cost US$ 10 000 for the complete conversion of the original scanning spectrometer into the mutidetection PDA system (including the photodiode array itself, boards, dispersion system, grating, supports, microcomputer and interface).

The entire conversion was carried out by M.F.P. as part of her doctoral work, and it did not require specialized electronic proficiency. The software and interface were adaptations of those presented in ref. [14]. Given the means, it is estimated that a dedicated researcher could accomplish the conversion, including calibration and testing, in about two months.

### Achnowledgements

The research reported here was partially supported by funds granted by the Brazilian agencies FACEPE, FINEP and PADCT. Scholarships granted by CNPq (PhD for M.F.P. and research for B.B.N., C.P and M.C.U.A.) are also gratefully acknowledged, and thanks are due to SPECTRO Analytical Instruments for providing the optical fibre used in this work.

### References

1. Noble, D., 1994, *Analytical Chemistry*, **66**, 105A.
2. Olesik, J. W., 1991, *Analytical Chemstry*, **63**, 12A.
3. Busch, K. W. and Busch, M. A., 1990, *Multielement Detection System for Spectrochemical Analysis* (New York: John Wiley & Sons).
4. Busch, K. W., Malloy, B. and Talmi, Y., 1979, *Analytical Chemstry*, **51**, 670.
5. Talmi, Y., 1975, *Analytical Chemistry*, **47**, 658A.
6. McGeorge, S. W. and Salin, E. D., 1985, *Spectrochimica Acta*, **40B**, 435.
7. McGeorge, S. W. and Salin, E. D., 1985, *Spectrochimica Acta*, **40B**, 1039.
8. Karanassios, V. and Horlick, G., 1986, *Applied Spectroscopy*, **40**, 813.

9. Levy, G. M., Quaglia, A., Lazure, R. E. and McGeorge, S. W., 1987, *Spectrochimica Acta*, **42B**, 341.
10. Brushwyler, K. R., Furuta, N. and Hieftje, G. M.,1990, *Talanta,* **37**, 23.
11. Winge, R. K., Fassel, V. A. and Edelson, M. C., 1988, *Spectrochimica Acta*, **43B**, 85.
12. Lepla, K. C. and Horlick, G., 1989, *Applied Spectroscopy*, **43**, 1187.
13. Brett, L., Stahl, R. G. and Timmins, K. J., 1989, *Journal of Analytical Atomic Spectrometry*, **4**, 333.
14. Belatto, C. R., Rohwedder, J. R., Raimundo, Jr, I. and Pasquini, C., 1996, *Journal of Automatic Chemistry*, **18**, 7.
15. Bilhorn, R. B., Sweedler, J. V., Epperson, P. M. and Denton, M. B., 1987, *Applied Spectroscopy*, **41**, 1125.
16. Epperson, P. M., Sweedler, J. V., Bilhorn, R. B., Sims, G. R. and Denton, M. B., 1988, *Analytical Chemistry*, **60**, 327A.
17. Bilhorn, R. B. and Denton, M. B., 1989, *Applied Spectroscopy*, **43**, 1.
18. Barnard, T. W., Crockett, M. I., Ivaldi, J. C., Lundberg, P. L., Yates, D. A., Levine, P. A. and Sauer, D. J., 1993, *Analytical Chemistry*, **65**, 1231.
19. Barnard, T. W., Crockett, M. I., Ivaldi, J. C. and Lundberg, P. L., 1993, *Analytical Chemistry*, **65**, 1225.
20. Van Veen, E. H. and Loos-Vollebregt, M. T. C., 1990, *Spectrochimica Acta*, **45B**, 313.
21. Ivaldi, J. C., Tracy, D., Barnard, T. W. and Slavin, W., 1992, *Spectrochimica Acta*, **47B**, 1361.
22. Glick, M., Brushwyler, K. R. and Hieftje, G. M., 1991, *Applied Spectroscopy*, **45**, 328.
23. Zagatto, E. A. G., Jacintho, A. O., Krug, F. J., Reis, B. F., Bruns, R. E. and Araújo, M. C. U., 1983, *Analytical Chimica Acta*, **145**, 169.
24. Luan, S., Pang, H. and Houk, R. S., 1995, *Spectrochimica Acta*, **50B**, 791.
25. Zhang, Z., Piao, Z. and Zeng, X., 1993, *Spectrochimica Acta*, **48B**, 403.
26. Wirsz, D. F. and Blades, M. W., 1986, *Analytical Chemistry*, **58**, 51.
27. EG & G Reticon, 1992, *Operation and Aligment Procedures for RC1000/10001.*
28. EG & G Reticon, 1992, S-series Solid State Line Scanners 128, 256, 512 and 1024 Elements.
29. RS Data Library, 1988, Peltier Effect Heat Pumps, Catalogue 9192.
30. Malcome-Lawes, D. J., 1987, *Laboratory Microcomputer*, **6**, 16.
31. Malcome-Lawes, D. J., 1987, *Laboratory Microcomputer*, **6**, 122.
32. Pimentel, M. F., Barros Neto, B., Saldanha, T. C. B. and Araújo, M. C. U., *Journal of Automatic Chemistry*, in press.
33. Draper, N. R. and Smith, H., 1981, *Applied Regression Analysis* (New York: John Wiley and Sons).
34. Wirsz, D. F., Browne, R. J. and Blades, M. W., 1987, *Applied Spectroscopy*, **41**, 1383.
35. Pimentel, M. F., Barros Neto, B., Araújo, M. C. U. and Pasquini, C., *Spectrochimica Acta Part B,* in press.

